# Customized Peptide Biomaterial Synthesis via an Environment-Reliant Auto-Programmer Stigmergic Approach

**DOI:** 10.3390/ma11040609

**Published:** 2018-04-16

**Authors:** Ravindra V. Badhe, Pradeep Kumar, Yahya E. Choonara, Thashree Marimuthu, Lisa C. du Toit, Divya Bijukumar, Dharmesh R. Chejara, Mostafa Mabrouk, Viness Pillay

**Affiliations:** 1 Wits Advanced Drug Delivery Platform Research Unit, Department of Pharmacy and Pharmacology, School of Therapeutic Sciences, Faculty of Health Sciences, University of the Witwatersrand, Johannesburg, 7 York Road, Parktown 2193, South Africa; badheravi2@gmail.com (R.V.B.); pradeep.kumar@wits.ac.za (P.K.); yahya.choonara@wits.ac.za (Y.E.C.); thashree.marimuthu@wits.ac.za (T.M.); lisa.dutoit@wits.ac.za (L.C.d.T.); divyaraniv@gmail.com (D.B.); dharmeshchejara@gmail.com (D.R.C.); mostafamabrouk.nrc@gmail.com (M.M.); 2 Department of Refractories, Ceramics and Building Materials, National Research Centre, 33 El-Bohouth St. (former El-Tahrir St.), Dokki, Giza P.O. 12622, Egypt

**Keywords:** stigmergy, peptide biomaterial synthesis, self-organizing peptides, effect of temperature on protein digestion

## Abstract

Stigmergy, a form of self-organization, was employed here to engineer a self-organizing peptide capable of forming a nano- or micro-structure and that can potentially be used in various drug delivery and biomedical applications. These self-assembling peptides exhibit several desirable qualities for drug delivery, tissue engineering, cosmetics, antibiotics, food science, and biomedical surface engineering. In this study, peptide biomaterial synthesis was carried out using an environment-reliant auto-programmer stigmergic approach. A model protein, α-gliadin (31, 36, and 38 kD), was forced to attain a primary structure with free –SH groups and broken down enzymatically into smaller fragments using chymotrypsin. This breakdown was carried out at different environment conditions (37 and 50 °C), and the fragments were allowed to self-organize at these temperatures. The new peptides so formed diverged according to the environmental conditions. Interestingly, two peptides (with molecular weights of 13.8 and 11.8 kD) were isolated when the reaction temperature was maintained at 50 °C, while four peptides with molecular weights of 54, 51, 13.8, and 12.8 kD were obtained when the reaction was conducted at 37 °C. Thus, at a higher temperature (50 °C), the peptides formed, compared to the original protein, had lower molecular weights, whereas, at a lower temperature (37 °C), two peptides had higher molecular weights and two had lower molecular weights.

## 1. Introduction

Peptide biomaterial synthesis is being revolutionized with advances in peptide synthesis mimicking natural protein synthesis to produce long and short peptide fragments in large quantities [1]. Many researchers have reported the use of short designed peptide molecules that self-assemble to form well-ordered nanostructures such as spheres, cylinders, tubes, and many other morphologies. These structures exhibit several desirable qualities for drug delivery, tissue engineering, cosmetics, antibiotics, food science, and biomedical engineering. Use of these structures in drug delivery involves self-assembling peptide (SAP) forming nanoparticles as target specific drug delivery vehicles. In tissue engineering, they are extensively studied as ECM mimicking nanofibers/nanostructures, and along with stem cells they are used to study enhanced tissue regeneration. These self-assembled peptide nanostructures can act as bio-compatible materials and be coated on implantable devices like stents, metallic screws, etc. In the cosmetics and food industries, these SAPs are used in hydrogel development for various applications. In addition to these applications, SAPs are also used in the development of peptide hormones, lipopeptides, and peptide semiconductors [2,3,4,5]. Though self-assembling short designed peptides to synthesize nanostructures is a very appealing process, a host of pure amino acids and a significant amount of time is required to synthesize a large quantity of short peptides, which makes it costly and time-consuming. The authors have observed that the enzymatic digestion of the large-molecular-weight proteins generates smaller-molecular-weight fragments of different lengths. It is a mixture of long and short peptides and can self-organize to form nano- or micro-structures with similar desirable qualities as that of self-assembled peptides. The authors have also observed that the self-organization of different peptide fragments generated by protein digestion is an environment-dependent auto-programmer stigmergy phenomenon.

### Stigmergy

The stigmergic principles have been used to analyze ever-widening range of self-organizing activities in domains ranging from social insects, human society, robotics, and social media to individual cognition and internet-supported collaboration. The term “stigmergy” was introduced in 1959 by a French biologist, Pierre-Paul Grassé, with reference to termite behavior. It is derived from the Greek words stigma (“mark” or “sign”) and ergon (“work” or “action”) [6]. Stigmergy is defined as a complex and intelligently structured “self-organization”. The structures formed via stigmergy do not require any planning, control, or communication between agents. Stigmergy accounts for the self-organization mechanism of simple agents that are devoid of any memory or intelligence. It is based on the mechanism of indirect coordination, in which the trace left by an action in a medium stimulates the next action, by the same or a different agent. This leads to a strengthening and assembling of agents over each other and the development of a complex and intelligent structure [7,8]. In this work, it was attempted to prove that the biochemical and chemical reactions may also be governed by stigmergic principles. 

## 2. Materials and Methods 

### 2.1. Materials

α-Gliadin, α-chymotrypsin, 6M urea, and DL-dithiothreitol (DTT) for protein digestion; 40% acrylamide/bis-acrylamide solution, *N*,*N*,*N*′,*N*′-tetramethylethylenediamine (TEMED), Tris buffer, ammonium persulfate (APS), Coomassie Brilliant Blue G-250 stain for SDS-PAGE, and Pur-A-Lyzer Dialysis Kits (3.5 kDa MWCO) were purchased from Sigma-Aldrich (Cleveland, OH, USA). All other reagents used were of analytical or equivalent grade. Water used for reagent preparation and dialysis was Milli-Q water.

### 2.2. Methods

Any process involving reactions (chemical, biological, or enzymatic) between reactants (agents) occurs spontaneously or under the influence of external factors (environment), ultimately yielding a product. If the system is simple involving a reaction between two agents in the environment leading to a product, it can be understood by the laws and principles of chemistry and thermodynamics. If the system is complex involving multiple agents in controlled (programmed) environment, interacting in steps, and finally forming a product without external interference; it can be referred to as a stigmergy process. The digestion or breakdown of the protein into peptide fragments with the help of a proteolytic enzyme and the related self-organization of the peptide fragments in the programmed environment to form a new peptide-based biomaterial was chosen as the biomaterial synthesis process to be compared with the stigmergic process.

To prove this hypothesis, α-gliadin was chosen as the model protein. α-Gliadin is isolated from wheat. The molecular weight of gliadin ranges between 31 and 38 kD, and it contains 266 amino acids and 3 disulfide linkages (126–156, 157–247, and 169–255). It is a positively charged water-insoluble protein [9,10]. When α-gliadin was digested with the help of α-chymotrypsin, the peptide fragments generated during this process form new peptide molecules by self-organization. This self-organization was governed by the factors of reaction environment such as temperature and termed as an environment-reliant auto-programmer stigmergic process. 

#### 2.2.1. The “*In Solution*” Digestion Protocol for Peptide Biomaterial Synthesis by the Stigmergic Process 

**Figure 1:**
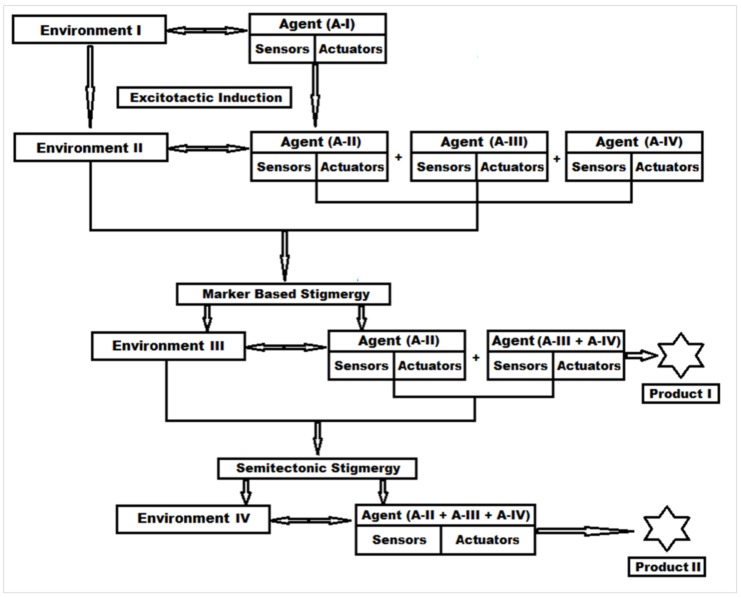
Schematic representation of peptide biomaterial synthesis by environment-reliant auto-programmer stigmergy.

The in-solution digestion of protein to synthesize new peptide-based biomaterials based on stigmergic principles can be understood from Figure 1. The proteolytic digestion of α-gliadin protein (Agent A-I) with α-chymotrypsin was carried out as per the method given by Vermachova et al. [11], with modifications. Briefly, α-gliadin sample (100 mg) was dissolved in approximately 1 mL of 6.0 M urea in a 10 mL glass polytop bottle and pH was adjusted to 9. Once all the α-gliadin was dissolved, the reaction mixture was reduced with DTT (5 mM) for 20 min at 37 °C in the dark (Environment I). The dissolution of α-gliadin, which is a hydrophobic protein in 6 M urea, causes the 3D structure of protein (Sensors) to unfold [12]. The unfolded structure exposes the disulfide bonds (Actuators) hidden in the inner core, and DTT, being a reducing agent, breaks it down to give primary structure of α-gliadin. This mixture was diluted up to 6 mL with Milli-Q water, and α-chymotrypsin, in a α-chymotrypsin/α-gliadin ratio of 1:30 (Environment II), was added to it, which broke down the primary structure of α-gliadin into smaller fragments (Agents II, III, and IV) based on the specific amino acid sequence (Sensors). These fragments are now composed of free –NH and –COO terminals, charged side chains, a free –SH group, and other forces (Actuators), which act as markers and initiate these fragments to self-organize. The 6 mL solution was then divided equally in two glass polytop bottles and incubated at two different temperatures (37 and 50 °C) (Environment III) for 24 h with constant stirring. This new environment influenced the self-organization of fragments leading to the formation for Product 1 (greater peptide fragments) by a marker-based stigmergy process. After 24 h of incubation, the contents of the polytop glass bottles were transferred to a dialysis membrane and dialyzed in Milli-Q water (Environment IV) for 48 h. During this time, the suppressed forces (Actuators) of non-organized peptides in Environment III are activated due to the reduction in urea and DTT concentration in Environment IV. This activation of the unreacted peptides then interact with larger peptide fragments formed as Product 1 and a build greater peptide, Product 2, a by semitectonic stigmergy process.

The proteolytic digestion method was slightly modified by avoiding the addition of an alkylating agent. The alkylating agent may react with free sulfhydryl groups of cysteine residues to form a complex, which cannot be reoxidized to form disulfide bonds. Thus, this step might have reduced the number of actuators present in the agent.

The in-solution digested protein (at 37 and 50 °C) were dialyzed separately using Pur-A-Lyzer Dialysis Kits (3.5 kDa MWCO) against Milli-Q water and lyophilized. The lyophilized matter contains a mixture of peptides that can be analyzed further by SDS-PAGE and MALDI-TOF-MS with further purification.

#### 2.2.2. Characterization of Peptide Biomaterial Synthesized by the Stigmergic Process 

##### Molecular Weight Determination of Stigmergized Peptides using SDS-PAGE

The protein composition of gliadin and self-organized peptide samples were analyzed using sodium dodecyl sulfate-polyacrilamide gel electrophoresis (SDS-PAGE) performed according to conditions described by Laemmli (1970) [13]. Briefly, the 12% separating gels and 5% stacking gels were cast in vertical electrophoretic units (Mini-PROTEAN, Bio-Rad Laboratories, Hercules, CA, USA). Samples were diluted with the sample buffer (without β-mercaptoethanol) in a ratio of 1:2 (v/v). These samples were then heated at 90 °C for 5 min and cooled at room temperature. Gliadin fractions equivalent to 5 and 7 µg were loaded in two wells. Similarly, the self-organized peptides at 37 and 50 °C were loaded at 5, 7, and 10 µg and 5, 7, 10, and 12 µg concentrations, respectively, on separate gels. Gels were run at 100 mA for 3 h, and stained with Coomassie blue staining. The molecular weights of the peptides were estimated using ultra-low and low-range molecular-weight standards ranging from 1.1 to 26.6 kD and from 6.5 to 66 kD (Sigma-Aldrich, Cleveland, OH, USA), respectively. To analyze possible high-molecular-weight stigmergized products, bovine serum albumin (66 kD, SDS-PAGE marker from Sigma-Aldrich, Cleveland, OH, USA) was added to an ultra-low molecular-weight standard. The protein bands on the Coomassie blue stained gel were analyzed using IQuant Capture 300 software (GE Healthcare, Buckinghamshire, UK). The electrophoresis was performed in triplicate to authenticate the self-organized peptides. 

##### Molecular Weight Confirmation of Stigmergized Peptides using MALDI-TOF Analysis

The lyophilized matter of in-solution digested protein (at 37 and 50 °C) contains a mixture of peptides and residual salts that were removed using a C18 spin column (Thermo Fisher Scientific, Waltham, MA, USA). First, the column was washed with acetonitrile and then rinsed and saturated with 0.1% TFA. The peptide solution was loaded onto the column and rinsed 2–3 times with 0.1% TFA. Subsequently, the column was rinsed three times with water to remove the TFA that might interfere with mass analysis of peptides. The peptides were then eluted from the column using MALDI-TOF-MS/MS elution solution (Gundry et al.) [14] and collected in a clean tube and used for MALDI-TOF analysis.

The molecular weight determination of the purified stigmergized peptides were performed using Autoflex Smartbeam III MALDI-TOF/TOF system (Bruker Daltonics Inc., Billerica, MA, USA). Approximately 10 pmol of samples were mixed with the matrix consisting of 10 mg/mL alpha-cyano-4-hydroxy-cinnamic acid (CHCA) dissolved in 50/50 acetonitrile/ethanol, spotted on the target plate, evaporated to dryness, and then detected in the linear mode MALDI-TOF using a mass range of 5000–30,000 *m*/*z*. The laser power was set at 60%. An average of 1000 shots was used to acquire the spectra. 

##### Secondary Structure Analysis of Stigmergized Peptides 

The stigmergized peptide bands resolved in SDS-PAGE and stained with Coomassie blue dye, were isolated from a gel slab as per the method described by Purcell et al. [15], lyophilized, deuterated with D_2_O, and analyzed for absorbance using FTIR over a range of 1700–1600 cm^−1^ at room temperature with a 4 cm^−1^ resolution and 20 scans/sample scanning rate (PerkinElmer spectrum 100 FT-IR Spectrometer with ATR sampling accessory and the PerkinElmer spectrum software package (Perkin Elmer, ‎Waltham, MA, USA). 

##### Morphology of Stigmergized Peptides

The morphology of the original α-gliadin and isolated stigmergized peptides were observed using a scanning electron microscope (SEM) (Phenom-FEI, Eindhoven, The Netherlands). The lyophilized powder of α-gliadin and isolated stigmergized peptides were spread and fixed with double-sided carbon tape onto aluminum stubs. Samples were then coated with gold in argon plasma with a sputter coater (SPI Module Sputter Coater, West Chester, PA, USA).

##### Predicted Self-Organization of Stigmergized Peptides

The stigmergic self-organization of peptide fragment based on the molecular weight provided by SDS-PAGE and secondary structure provided by FTIR can be predicted using modeling software HyperChemTM 8.0.8 (Hypercube Inc., Gainesville, FL, USA) and ChemBio3D ultra 11.0 (CambridgeSoft Corporation, Cambridge, UK). The α-gliadin protein contains 266 amino acid residues with three disulfide linkages [9,10]. Treatment with 6M urea and DTT converted the 3D structure of the protein into a primary structure with the following amino acid sequence:


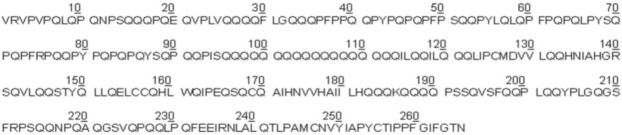


This primary structure of protein was broken down to 10 possible peptide fragments by α-chymotrypsin (PeptideMass-ExPASy bioinformatics resource portal). The fragments with their molecular weight are presented in Table 1. The fragments with cysteine amino acid were then stigmergized to synthesize new peptide molecule.

## 3. Results

### 3.1. Stigmergy-Based Mechanism of Protein Digestion and New Peptide Biomaterial Synthesis

With an understanding of the stigmergic classification (supplementary data), protein digestion reaction can be categorized as a qualitative-marker-based stigmergic process. This process is based on the influence of the environment on the agent. Each agent has its own sensors and actuators that act as markers, and these sensors and actuators are activated by the respective environment. To better understand this environment-reliant auto-programmer stigmergy in the peptide biomaterial synthesis, the reactants and the reactions conditions were assigned as agents and environments.

#### 3.1.1. Step 1—Engineering “Agent-State” to “Agent-Dynamic”

In the correlation of the protein digestion reaction to form a new biomaterial, the first step is to understand the agent-state and the environment’s state. In this enzymatic reaction, the protein to be digested is in its 3D form (tertiary structure) and acts as an agent-state, and the medium in which the protein is dissolved (in this case urea solution) acts as environment’s state (Figure S2).

**Figure 2:**
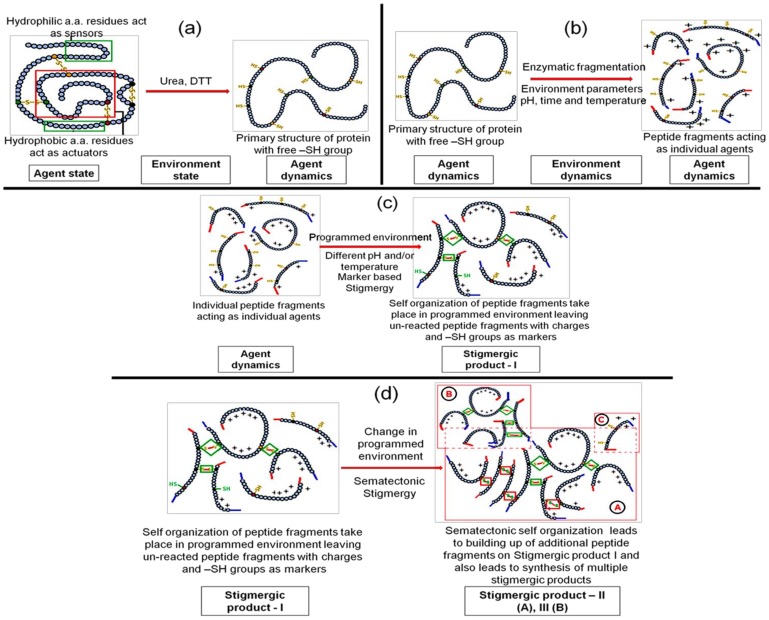
Stigmergy-based mechanism of protein digestion and new peptide biomaterial synthesis. (**a**) Engineering agent-state to agent-dynamic; (**b**) Engineering the environment’s dynamic and multi-agent systems; (**c**) Marker-based stimulus to generate stigmergic product; (**d**) Sematectonic emergence of stigmergic products.

Agent-state, as per Pannequin et al. [16], should contain sensors and actuators (Figure S1). A tertiary structure (3D) is the most stable structure and generally resist any change. There are certain regions (hydrophilic amino acids) in the tertiary structure that act as sensors and help urea and water molecules to attach to them by hydrogen bonds. The core of the tertiary structure (3D) is mainly comprised of hydrophobic amino acids that act as actuators. These actuators slowly open the complete tertiary structure as the sensor amino acids take up urea water with hydrogen bonds. The mechanism behind this phenomenon involves the wetting and electrostatic binding property of the urea molecule, which converts the hydrophobic amino acids to hydrophilic charged amino acids, which in turn leads to an opening of the tertiary protein molecule, known as the “outside in” phenomenon or the denaturation of protein [17,18]. Once the denaturation is complete and the protein tertiary structure is open; the –S–S– bonds are exposed to the environment state that consists of dithiothreitol (DTT). DTT breaks the –S–S– bonds and converts the tertiary structure of the protein to a primary structure, which acts as the agent-dynamic (Figure 2a). This transition takes place in an environment state that is programmed with stable parameters such as pH, temperature, and time.

#### 3.1.2. Step 2—Engineering the Environment’s Dynamic and Multi-Agent Systems

Once the agent-dynamic is achieved, the environment’s state can be modified to environment dynamics by adding a proteolytic enzyme. This enzyme cuts the primary structure amino acid sequence of protein at specified locations to yield peptide fragments. These peptide fragments contain active –SH groups, terminal –COOH and –NH_2_ groups, amino acids residues with hydrogen-bond-forming side chains, and electrostatic and ionic charges. Thus, these peptide fragments become multi-agent-dynamic and become a multi-agent system (Figure 2b). In the environment’s dynamic, the optimized pH and temperature for the proteolytic enzyme along with the concentration and reaction time need to be maintained to obtain the multi-agent-dynamic system.

#### 3.1.3. Step 3—Marker Based Stimulus to Generate Stigmergic Product

The multi-agent-dynamic system consists of sensors in the form of electrostatic forces on molecules and actuators in the form of free –SH groups (Figure S3). If there is any change in the environment’s dynamic parameters (pH, temperature, etc.), electrostatic forces on the molecule sense it and activate the –SH groups. Activated –SH groups start forming covalent bonds with a nearby –SH group to generate –S–S– bonds with other peptide fragments to generate a new complex peptide structure. This spontaneous self-organization of peptide fragments is a stigmergic process and hence the product is a stigmergic product (Figure 2c). 

#### 3.1.4. Step 4—Sematectonic Emergence of Stigmergic Products

The self-organization in Step 3 leaves a trail of some unreacted peptide fragments in the environment. These fragments contain –SH groups and are influenced by electrostatic forces. The modification in the environment stimulates the system for the next self-organization reactions, which are build-up reactions known as sematectonic self-organization, as suggested by Giuggiolia et al. [19]. This process of continuous synthesis led to the build-up of additional peptide fragments on Stigmergic Product I and to the synthesis of multiple stigmergic products (Figure S3).

The production of such useful stigmergic entities (peptide biomaterials) can be achieved by carefully programming the environment dynamics for pH, temperature, and time of the reaction (Figure 2d). Thus, in conclusion, the symmetric 3D protein molecule was broken down into smaller peptide clusters of scattered symmetry with lower entropy, which conformed to the stable self-organization of the peptide biomaterials, as organization is inversely proportional to symmetry (Figures S4 and S5) [20,21].

### 3.2. Characterization of Synthesized Peptide Biomaterial 

#### 3.2.1. Molecular Weight Analysis of Stigmergized Peptides

**Figure 3:**
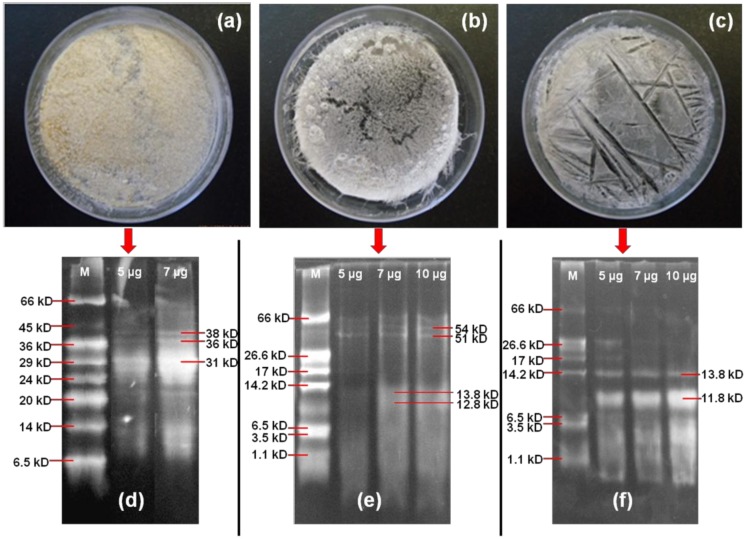
Physical state and molecular weight analysis of α-gliadin (**a**,**d**); the self-organized peptide biomaterials at 37 °C (**b**,**e**); and the self-organized peptide biomaterials at 50 °C (**c**,**f**).

The physical state and molecular weight analysis of gliadin and self-organized peptide biomaterials at different environmental conditions (37 and 50 °C) are shown in Figure 3. The environment-reliant effect on the morphology (Figure 3a–c) can be clearly observed for gliadin (a), stigmergized peptide at 37 °C (b), and stigmergized peptide at 50 °C (c). The morphology change from powdered through semi-structured to structured (from Figure 3a–c) clearly suggested the formation of a new, well organized peptide at 50 °C (which did not occur at 37 °C). Thus, the peptide fragments self-organized differently, and this self-organization was governed by the environmental conditions [22,23].

The molecular weight analysis (Figure 3d–f) of gliadin, the self-organized peptide biomaterials at 37 °C, and the self-organized peptide biomaterials at 50 °C clearly suggested the synthesis of new peptide biomaterials with different peptide molecular weights compared to the original α-gliadin protein. The molecular weight of α-gliadin (d) was observed in the range of 31–38 kD in SDS-PAGE analysis, while the self-organized peptide biomaterials at 37 °C (e) showed the molecular weights approximately at 54, 51, 13.8, and 12.8 kD, and the self-organized peptide biomaterials at 50 °C (f) showed the molecular weights approximately at 13.8 and 11.8 kD. The peptide fragments self-organized to give two high-molecular-weight proteins and two low-molecular-weight peptide at 37 °C, whereas at 50 °C the peptide fragments self-organized to give two low-molecular-weight fragments. These observations suggested that the chymotryptic peptide fragments self-organized differently under the influence of different temperatures. It also suggested that the reactions took place during digestion. The self-organization was spontaneous. Thus, the self-organization mechanism of simple agents that were devoid of any memory or intelligence or even awareness of each other was explained.

#### 3.2.2. Molecular Weight Confirmation of Stigmergized Peptides

**Figure 4:**
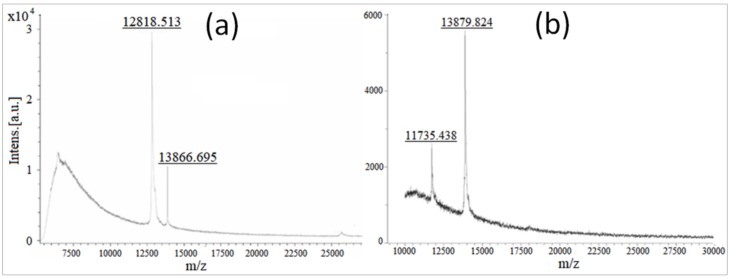
MALDI-TOF analysis of self-organized peptide biomaterials. (**a**) Spectra of *m*/*z* 12,818.513/13,866.695 peptides synthesized at 37 °C. (**b**) Spectra of *m*/*z* 11,735.438/13,879.824 peptides synthesized at 50 °C.

The chymotryptic breakdown of gliadin protein generated specific peptide fragments and stigmergic rearrangements of these peptide fragments at specific environments (37 and 50 °C) led to new peptide molecules with molecular weights that were higher and/or lower than the original gliadin protein. The peptide mass fingerprinting of stigmergized peptides were carried out by MALDI-TOF-MS. The spectrum of identified peptide masses was unique for a specific environment (either 37 or 50 °C) and thus acted as a mass fingerprint. As shown in Figure 4, the stigmergized peptides at 37 °C showed two peaks in MALDI-TOF at *m*/*z* 12,818 and 13,866, whereas the stigmergized peptides at 50 °C showed peaks at *m*/*z* 11735 and 13879 in the scanning range of *m*/*z* 0–30,000. These observations matched closely with the SDS-PAGE observations. 

#### 3.2.3. Secondary Structure Analysis of Stigmergized Peptides 

**Figure 5:**
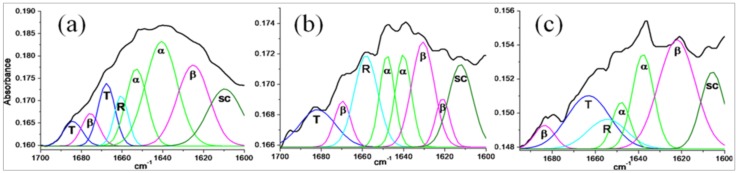
Secondary structure analysis from FT-IR spectra: gliadin (**a**); 11.8 kD isolated fragment (**b**); and 13.8 kD isolated fragment (**c**).

The FT-IR spectra for secondary structure analysis of gliadin (a) and isolated fragments (13.8 and 11.8 kD) of self-organized peptide biomaterials (b and c) are given in Figure 5 and probable secondary structures (in %) are given in Table 2. The secondary structure of α-gliadin matched closely with the reported secondary structures [24], while secondary structures of 11.8 and 13.8 kD peptides were completely different from α-gliadin and from each other. These observations suggested that the 11.8 and 13.8 kD peptide were new self-organized peptides. 

#### 3.2.4. Morphology of Stigmergize Peptides

**Figure 6:**
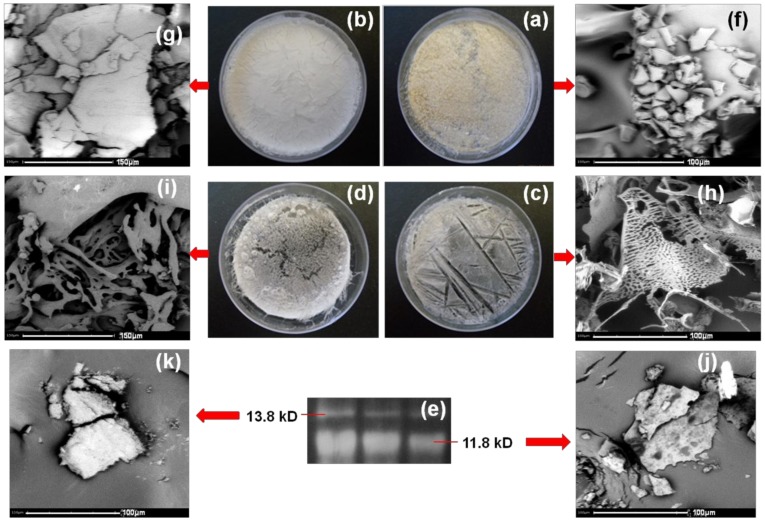
Morphology of α-gliadin (**a**,**f**); α-gliadin dissolved in urea (**b**,**g**); α-gliadin digested at 50 °C (**c**,**h**); α-gliadin digested at 37 °C (**d**,**i**); and isolated self-organized peptides (**e**) with molecular weights of 11.8 (**j**) and 13.8 kD (**k**).

Figure 6 showcase different morphologies of α-gliadin (a and f), α-gliadin dissolved in urea (b and g), α-gliadin digested at 50 °C (c and h), α-gliadin digested at 37 °C (d and i), and isolated self-organized peptides with molecular weights of 11.8 (j) and 13.8 kD (k). It was observed that α-gliadin digested at 50 °C (h) had a porous wafer structure, while α-gliadin digested at 37 °C (i) had a broken moss filament structure. The self-organized peptide of 11.8 kD (j) displayed a thin-film-like structure while peptide of 13.8 kD (k) had a cuboidal morphology. All these structures were very much different from the original α-gliadin protein (f). These well characterized and morphologically distinct shapes of the peptides provided evidence of the self-organization of peptides that were complex but seemingly intelligent structures.

#### 3.2.5. Predicted Self-Organization of Stigmergize Peptides

**Figure 7:**
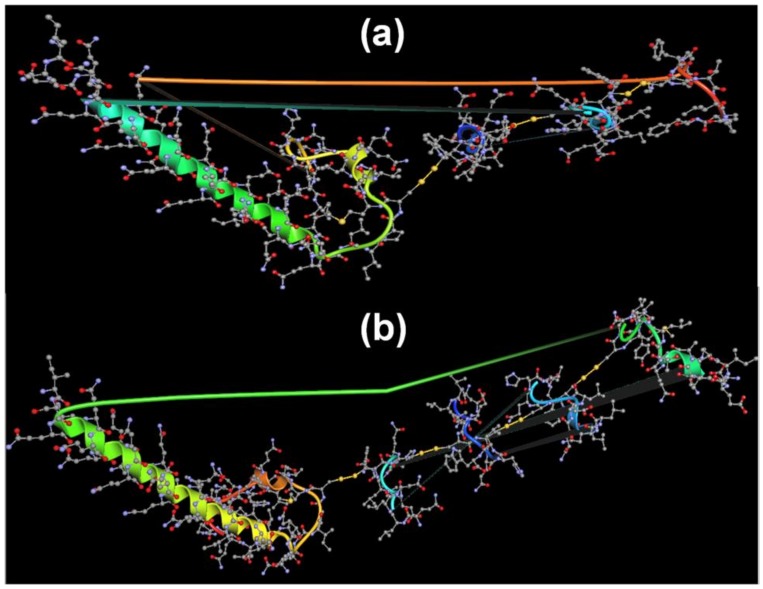
Predicted 3D structure of stigmergized peptides of (**a**) 11.8 and (**b**) 13.8 kD.

The self-organization of the peptide fragments (Table 1) can be predicted based on the molecular-weight data provided by MALDI-TOF analysis. Based on the MALDI-TOF molecular weights, the possible –S–S– bonding between one Fragment 1 peptide, two Fragment 9 peptides, and one Fragment 10 peptide may have led to the synthesis of an 11.8 kD peptide, and the –S–S– bonding between one Fragment 1 peptide, three Fragment 9 peptides, and one Fragment 6 peptides may have led to the synthesis of a 13.8 kD peptide. Thus, it can be predicated that Fragment 9 acted as the linker between terminal fragments as it contained two cysteine residues. The secondary structure predictor tool (PEP-FOLD3, RPBS, INSERM UMR-S 973, Institut Universitaire de France (IUF), Paris, France) was used to predict the approximate secondary structure of the formed peptides, and the secondary structures of both peptides (the 11.8 kD peptide—57% helical structure, 33% extended structure, 10% coils—and the 13.8 kD peptide—56% helical structure, 32% extended structure, 12% coils) corroborated the observations from the FT-IR spectra-based secondary structure predictions. The predicted rearrangement of the fragments leading to 3D peptides is shown in Figure 7. Therefore, the formation of specific-molecular-weight peptide fragments at specific temperatures (13.8 and 11.8 kD at 50 °C/54, 51, 13.8, and 12.8 kD at 37 °C) can be related to the environment-reliant stigmergic process.

## 4. Conclusions

This study was carried out to correlate a biochemical reaction to stigmergic principles. From this study, it can be concluded that an enzymatic digestion reaction of a protein molecule can lead to the formation of new peptide biomaterials. These well characterized and morphologically distinct peptides were complex and seemingly intelligent structures. They were synthesized by efficient collaboration between extremely simple agents such as small peptide fragments, which lack any memory or even awareness of presence of each other, without the need for any planning, control, or even direct communication between them. It was also observed that this reaction was governed by temperature, which confirmed the potential role of environment towards the formation of the final peptide. These observations fit exactly within the characteristics and definitions of stigmergy. Thus, this study provided insights into an important mechanism inherent in complex biochemical reactions on the basis of stigmergic principles.

## Figures and Tables

**Table 1:** The peptide fragments generated after α-chymotrypsin digestion of α-gliadin.

Fragment	Mass	Position	Peptide Sequence
1	7345.1270	88–149	SQPQQPISQQQQQQQQQQQQQQQQQQILQQILQQQLIPCM DVVLQQHNIAHGRSQVLQQS TY
2	5158.8066	31–74	LGQQQPFPPQQPYPQPQPFPSQQPYLQLQPFPQPQLPYSQ PQPF
3	4136.6144	162–197	QIPEQSQCQAIHNVVHAIIL HQQQKQQQQPSSQVSF
4	3493.9273	1–30	VRVPVPQLQPQNPSQQQPQE QVPLVQQQQF
5	2363.5747	212–232	RPSQQNPQAQGSVQPQQLPQ F
6	2078.4291	233–250	EEIRNLALQTLPAMCNVY
7	1626.7918	75–87	RPQQPYPQPQPQY
8	1590.7556	198–211	QQPLQQYPLGQGSF
9	1513.7924	150–161	QLLQELCCQHLW
10	1121.3595	251–260	IAPYCTIPPF

**Table 2:** Predicted secondary structure of stigmergized peptides.

Protein/Stigmergized Peptides	Secondary Structure (%)			
	α-Helix (α)	β-Sheet (β)	β-Turns (T)	Disordered (R and SC)
**α-Gliadin**	39	22	17	22
**11.8 kD**	62	10	17	11
**13.8 kD**	64	06	20	10

Secondary structure of α-gliadin matched closely with the reported secondary structure by Wong et al. [24]. The secondary structures of newly formed peptides of 11.8 and 13.8 kD are predicted from FT-IR spectra as shown in Figure 5.

## Supplementary Materials

The following are available online at http://www.mdpi.com/1996-1944/11/4/609/s1. Figure S1: Internal structure of a stigmergic physical agent. Figure S2: Basic architecture of Stigmergy. Figure S3: Schematic representation of stigmergy proceeding by marker-based and sematectonic mechanism. Figure S4: Inverse relation of Symmetry and Organization. Figure S5: Emergence as a Subcategory of Self-Organization. Table S1: Classification of Stigmergy.
