# Supplementary material for: Customized Peptide Biomaterial Synthesis via an Environment-Reliant Auto-Programmer Stigmergic Approach
Source: Materials (Basel). 2018 Apr 16;11(4):609. doi: 10.3390/ma11040609 (PMC5951493; doi:10.3390/ma11040609)
Supplement: Supplementary file 1 [file materials-11-00609-s001.pdf]

Supplementary Materials: Customized Peptide Biomaterial Synthesis via an Environment Reliant, Auto-Programmer, Stigmergic Approach

Ravindra V. Badhe, Pradeep Kumar, Yahya E. Choonara, Thashree Marimuthu, Mostafa Mabrouk, Lisa C. du Toit and Pillay Viness

Understanding stigmergy– Stigmergic system consists of agents and their distinct environment.

Parunak [1] defined the Features of agents as,

1. Agent contains an 'internal state' which is not directly approachable to other agents.
2. Agent contains 'sensors' which allows it to access to some of the environment's variables.
3. Agent contains 'actuators' which allows it to vary some of the environment's variables.
4. A program or dynamics (it covers the current internal state and signals received and released by its sensors and actuators to changes its internal state) (Figure S1). Agents can't sense all signals generated in the environment and thus differ to distinguish, interact, and respond to the environment [2].

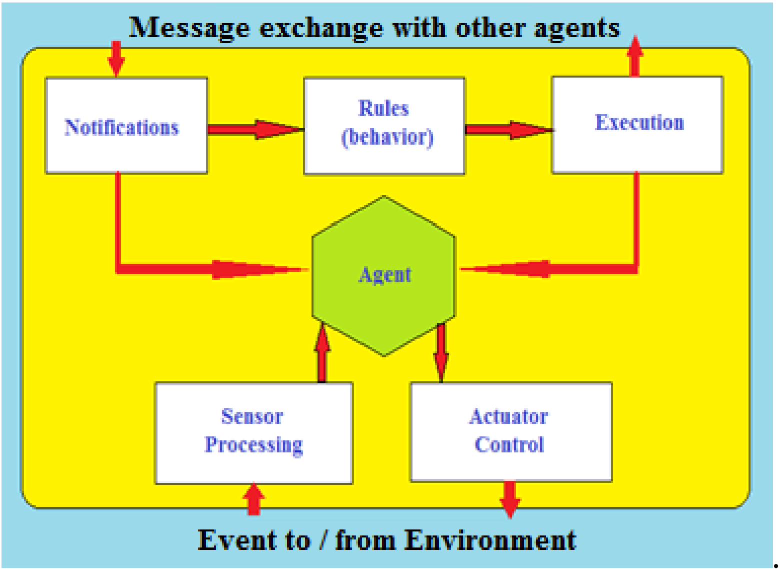

Figure S1. Internal structure of a stigmergic physical agent.

Parunak [3] defined features of the environment as:

1. The agents are always present in the structured 'environment' which provides ideal medium for interaction between agents. The agents instantly react to the changes occurred in the environment and change the way they interact with other agents with the change in the environment.
2. The conditions (program or dynamics) of the environment allow it to do certain information processing tasks on behalf of the system [4].

The 'agent' and 'environment' differ as the agents' internal state is hidden, thus each agent can act as self a sufficient, separate and confined unit. Whereas the environment is localized and contains multiple agents sharing defined environment space. These agents can interact with each other with their sensors and actuators in the environment [3]. Stigmergy is

manifested in the form of figure S2.

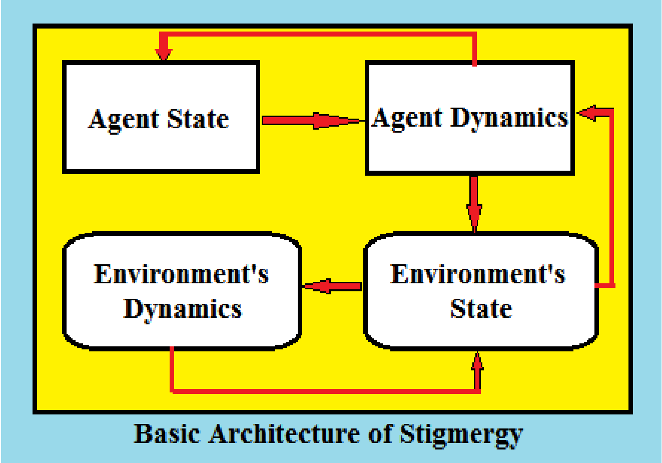

Figure S2. Basic architecture of Stigmergy.

In the reactions (chemical, biological or enzymatic), the agent state is comprised of the physical, chemical or physicochemical state as self. The series of events taking place in the surroundings (environment) of the agent which will influence the agent's state in the environment. This leads to change in both the agent and the environment leaving a trace of the agent, which serves as a signal for the next agent to process. Agent state and environment state will decide the response of an agent, thus each agent behaves separately (Figure S3). The products generated in stigmergic reaction completely depend upon interactions of agents under the influence of the environment state. The marker-based stigmergy works on the principal of first agent leaving the specific 'signature' (specilized marker) in the environment which signals and activates the other agent. The sematectonic stigmergy works on the principal that the agent responds to the signals (markers) present in the environment. Thus stigmergic cycle can complete as follows: first agent (being self sufficient unit or due to change in environment state) changes the environment by releasing marker (marker-based stigmergy). This marker in the environment is sensed by other agent that immediately responds to the marker (sematectonic stigmergy). This chain of reactions (cycle) continue if second sematectonic agent action leaves a mark in the environment.

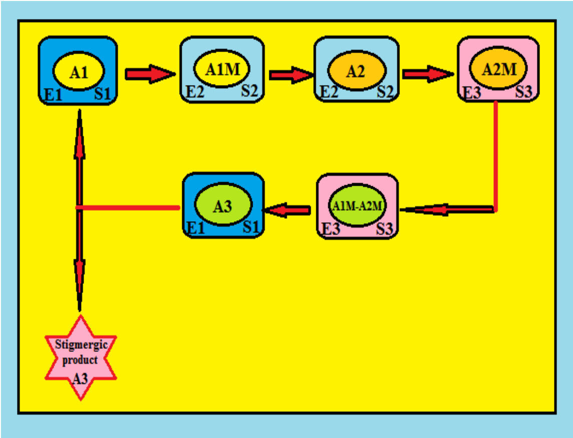

Figure S3. Schematic representation of stigmergy proceeding by marker-based and sematectonic mechanism.

When the agent A1 in environment E1 is stimulated by stimuli S1, it modifies itself to

A1M and generates stimuli S2 which modifies the environment to E2. This stimuli S2 then stimulates agent A2 and agent A2 get modified to A2M which intern modifies environment to E3 and generates stimuli S3. This stimulus S3 might stimulate another agent or might stimulate the self organization of A1M, A2M, A1, A2 to form a new agent A3 which is a stigmergic product. The new environment E1 with the stimuli S1 might start the cycle again from agent A1 or continues with the marker-based and sematectonic mechanism to form new agents [5].

Classification of Stigmergic Interaction

Parunak (2005a) categorized stigmergy in two groups, First group is sub-categorized as marker-based stigmergy and sematectonic stigmergy [6]. The second group is sub-categorized as quantitative stigmergy (signals are a single scalar analogous to a potential field) and qualitative stigmergy (signals form a set of discrete options) (Table S1) [7].

Table S1. Classification of Stigmergy.

	**Marker-based stigmergy**	**Sematectonic stigmergy**
**Quantitative**	Gradient following in a single pheromone field	Ant Cemetery Clustering
**Qualitative**	Decisions based on combinations of pheromones	Wasp Nest Construction

Marker based stigmergy - This hypothesis originated by observing certain social insects coordinate their actions with the use of pheromones. Insects generate different pheromones (markers) in different situations (environment). The selective pheromone provokes insects to take qualitative and/or quantitative decision. In a chemical reaction set, reactants act as stigmergic markers and solvent act as environment.

Sematectonic stigmergy - This hypothesis originated by observing some insect behaviours, where the environment generates certain signals which orients agents to work in coordination (the coordination of agents without markers) e.g. coordinated attack by ants if their cemeteries were compromised by invader [8]. This is a Sematectonic stigmergy quantitative decision.

Stigmergy have a number of attractive characteristics to be useful for different systems like Simplicity, Scalability, Robustness and Environmental integration.

Environmental integration is a very important characteristic as it involves interactions between agents in the environment. In this whole system of agents and environment, organization of system is inversely proportional to symmetry (Figure S4) and plays important role in self-organization of the agents in particular system [9]. In conventional control strategies, changes in environmental conditions change the final product. Whereas in stigmergic systems, change in environment conditions are used to break symmetrically arranged agents and re-organize them self to give new product [1,3].

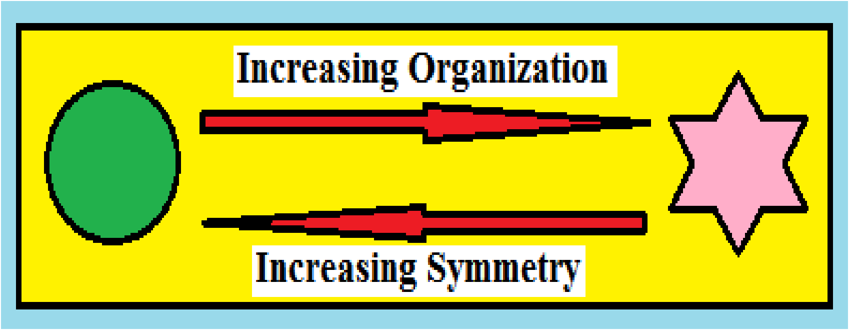

Figure S4. Inverse relation of Symmetry and Organization.

Emergent Behaviour - It is the development of new product with reduced entropy. It is the subcategory in self-organization (Figure S5). It states that, as the new product is less in entropy, it can be pathological. It also suggest to restrict conditions of interaction between agents and environment to ensure the newly formed product is beneficial or at least with no pathological properties [10].

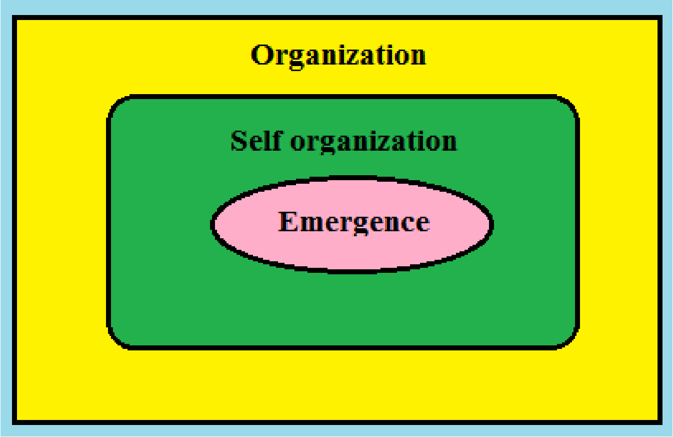

Figure S5. Emergence as a Subcategory of Self-Organization.

References

1. Van Dyke Parunak H., Expert Assessment of Human–Human Stigmergy. Analysis for the Canadian Defence Organization, Altarum Institute, Ann Arbor. MI (US), Oct. 2005.
2. Pannequin R., Thomas A. Stigmergy: Adesignpattern for Product-Drivensystems, 13th IFAC Symposium on Information Control Problems in Manufacturing, INCOM'2009, Moscou : Russian Federation, 2009.
3. Van Dyke Parunak H. Survey of Environments and Mechanisms for Human–Human Stigmergy." Paper presented at "Environments for Multi-Agent Systems II," Second International Workshop, Utrecht, the Netherlands, July 25. 2005.
4. Weyns D., Parunak H. V. D., Michel F., Holvoet T., and Ferber J. Multiagent Systems, State-of-the-Art and Research Challenges. In Proceedings of Workshop on Environments for Multi-Agent Systems (E4MAS 2004), New York, NY, Springer, 2004.
5. Giuggiolia L., Pottsa J. R., Rubensteind D. I., Levin S. A. Stigmergy, collective actions, and animal social spacing, PNAS, 2013, 110(42), 16904–16909.
6. Brueckner S. Return from the Ant: Synthetic Ecosystems for Manufacturing Control. Dr.rer.nat. Thesis at Humboldt University Berlin, Department of Computer Science, 2000. Available online: http://dochost.rz.hu-berlin.de/dissertationen/brueckner-sven-2000-06-21/PDF/Brueckner.pdf. (accessed on 16 April 2018)
7. Camazine S., Deneubourg J. L., Franks N. R., Sneyd J., Theraulaz G., Bonabeau E. Self-Organization in Biological Systems. Princeton, NJ, Princeton University Press, 2001.
8. Bonabeau E., Theraulaz G., Fourcassié V., Deneubourg J. L. The Phase-Ordering Kinetics of Cemetery Organization in Ants. Physical Review E, 4:4568-4571, 1998.
9. P. Ball. The Self-Made Tapestry: Pattern Formation in Nature. Princeton, NJ, Princeton University Press, 1996.
10. Wooldridge M. J. and Jennings N. R. Pitfalls of Agent-Oriented Development. In Proceedings of 2nd Int. Conf. on Autonomous Agents (Agents-98), Minneapolis, MN, 1998, 385-391.

© 2018 by the authors. Submitted for possible open access publication under the terms and conditions of the Creative Commons Attribution (CC BY) license (http://creativecommons.org/licenses/by/4.0/).
